# Induction of Invertebrate Larval Settlement; Different Bacteria, Different Mechanisms?

**DOI:** 10.1038/srep42557

**Published:** 2017-02-14

**Authors:** Marnie L. Freckelton, Brian T. Nedved, Michael G. Hadfield

**Affiliations:** 1 University of Hawaii, Kewalo Marine Laboratory, Honolulu, 96813, United States

## Abstract

Recruitment via settlement of pelagic larvae is critical for the persistence of benthic marine populations. For many benthic invertebrates, larval settlement occurs in response to surface microbial films. Larvae of the serpulid polychaete *Hydroides elegans* can be induced to settle by single bacterial species. Until now, only *Pseudoalteromonas luteoviolacea* had been subjected to detailed genetic and mechanistic studies. To determine if the complex structures, termed tailocins, derived from phage-tail gene assemblies and hypothesized to be the settlement cue in *P. luteoviolacea* were present in all inductive bacteria, genomic comparisons with inductive strains of *Cellulophaga lytica, Bacillus aquimaris* and *Staphylococcus warneri* were undertaken. They revealed that the gene assemblies for tailocins are lacking in these other bacteria. Negatively stained TEM images confirmed the absence of tailocins and revealed instead large numbers of extracellular vesicles in settlement-inductive fractions from all three bacteria. TEM imaging confirmed for *C. lytica* that the vesicles are budded from cell surfaces in a manner consistent with the production of outer membrane vesicles. Finding multiple bacteria settlement cues highlights the importance of further studies into the role of bacterial extracellular vesicles in eliciting settlement and metamorphosis of benthic marine larvae.

Larval recruitment to benthic marine communities is a critical process for population persistence, as well as for creating harvestable populations of mariculture species like clams, oysters and shrimp. The research described here focuses on the questions: what habitat-specific environmental cues ensure successful larval recruitment for benthic marine invertebrate species, and how do environmental cues act on larvae to bring about settlement and metamorphosis? Larvae of many benthic marine invertebrates settle on surfaces and metamorphose in response to complex biofilms. Evidence is accumulating that it is particular bacterial species residing in the biofilms that induce the larval response^1^. Biofilm- or bacteria-induced settlement has been shown for larvae of sponges^2^,^3^,^4^, cnidarians^5^,^6^, bryozoans^7^,^8^, molluscs^9^,^10^,^11^,^12^, annelids^13^, echinoderms^14^,^15^, crustaceans^16^,^17^ and urochordates^18^. The number of larval types recorded to settle and metamorphose in response to bacterial films is now so great as to suggest a nearly universal mechanism for both settlement induction and larval response, yet we know very little about either of these factors.

Extensive literature on the chemistry of settlement inducers was reviewed by Pawlik^19^ and Hadfield and Paul^20^. Surprisingly, little new information has been subsequently contributed to this area. A few studies employing a natural products chemistry approach have successfully identified small molecules important to the metamorphosis and settlement process. For example, a single small, non-polar bacterial metabolite, tetrabromopyrrole, was recently linked to partial or complete metamorphosis for some corals^21^,^22^. Conversely, the, polar compound histamine, produced by a marine alga, was found to be responsible for cueing settlement in an echinoid larva^23^. Numerous studies on the settlement of barnacle cyprid larvae focus on a “settlement-inducing protein complex” or SIPC secreted onto surfaces by conspecific barnacles and their cyprids^24^. Other studies on barnacle settlement also cite the requirement of bacterial films, even for the same barnacle species, *Amphibalanus amphitrite*^16^,^25^,^26^,^27^,^28^. These results may reflect the presence of multiple settlement cues for barnacle larvae^29^.

The serpulid polychaete *Hydroides* *elegans* has emerged as a useful model for studying bacterial induction of settlement and metamorphosis^13^,^30^. Larvae of *H. elegans* typically do not settle in the absence of a biofilm, and numerous biofilm bacterial species will induce their metamorphosis^31^,^32^. However, some – perhaps most – biofilm-bacterial species do not induce settlement in *H. elegans*. Furthermore, Huang and Hadfield^33^ demonstrated that larval response to bacteria in surface films is cell-density dependent. Among inductive bacterial species for larvae of *H. elegans* are strains of the broadly distributed genus *Pseudoalteromonas*. The same strains or others have been implicated in larval settlement of corals^34^, hydrozoans^35^ and echinoderms^14^.

A strongly inductive bacterium for larvae of *H. elegans, Pseudoalteromonas luteoviolacea*, has been investigated in depth to identify what elements are responsible for metamorphic induction in this larva. Huang and colleagues^36^ carried out random transposon mutagenesis on the bacterium and identified gene sequences that correlated with the inductive activity. Analysis of those gene sequences implicated elements of phage-tail proteins in the bacterial genome. A detailed analysis of the genome of *P. luteoviolacea* in the same region revealed a series of open reading frames encoding the complete structure of phage-tail elements similar to R-type pyocins from *Pseudomonas aeruginosa*^37^,^38^ and the defective prophage of *Serratia entomophila*^39^. In *P. aeruginosa* the structures are employed in inter-bacterial warfare where they are used to puncture the cell membranes of other, competing bacteria causing membrane depolarization and cell death^37^. In *S. entomophila* these structures have been associated with anti-grazing impacts on the grass grub *Costelytra zealandica*^39^. Pyocins were renamed as ‘tailocins’ by Gill and Young^40^ to try to simplify the terminology associated with these molecular machines, as well as, to reflect their isolation from unrelated bacterial strains. The term ‘tailocin’ was drafted because the genes transcribing the structure is derived from those of the T4-type phage tail assemblies^40^. The bactericidal activity of the original pyocins placed these structures among other antibacterial bacterial products termed bacteriocins, in particular high molecular weight toxins. Among them, the pyocin-like structures are labeled R-type bacteriocins. The term phage-like protein translocation structures (PLTSs) has also been applied to similar structures^41^,^42^. There is, however, currently no evidence for protein translocation in the phage-tail-derived structures, and to clearly distinguish between bacteriocins derived from phage–tail gene assemblies and bacteriocins that are small peptide toxins, the term tailocin will be used from here.

In seeking an understanding of the mechanism by which the tailocins of *P. luteoviolacea* induce larval settlement, Shikuma and colleagues^38^ discovered that the tailocins are assembled into complex and highly arranged structures. The arrays, labelled metamorphosis-associated contractile structures (MACs), were found to occur in only about 2% of cells in a biofilm. The question of how these arrays act on tubeworm larvae to induce their settlement and metamorphosis, however, remains unanswered. Do they simply puncture the larva in a specific excitable receptor causing the cell to depolarize and generate the cascade of events that transforms a ciliated, swimming larva into a sessile, tube-dwelling worm? Or do the tailocin clusters inject some morphogenetic product into larvae that acts in a hormone-like manner to bring about metamorphosis? And finally, is this a common mechanism to trigger metamorphosis in *H. elegans* by other unrelated bacterial strains?

Among the original biofilm-bacterial species identified by Huang and Hadfield^33^ that, in addition to *P. luteoviolacea*, induced larval settlement was another broadly distributed inductive marine bacterium, *Cellulophaga (Cytophaga) lytica*. Subsequently, two biofilm bacterial strains have been found to induce settlement in larvae of *H. elegans: Bacillus aquimaris* and *Staphylococcus warneri*. These two strains are Gram-positive, whereas both *P. luteoviolacea* and *C. lytica* are Gram-negative strains. The discovery that bacterial phylogeny is not correlated to metamorphic induction in *H. elegans* is not new^32^,^33^. The identification of these inductive Gram-positive bacteria, however, provided an opportunity to investigate the potential for a universal mechanism for settlement. Side-by-side preparations of bacterial products of *C. lytica, B. aquimaris* and *S. warneri* comparable to the same preparations from *P. luteoviolacea* were examined with transmission electron microscopy (TEM) to visually determine if tailocins were present, and, if so, were arranged in the complexes seen in *P. luteoviolacea*. Preparations of *C. lytica* were also examined with scanning electron microscopy (SEM) to verify the origin of the vesicles. In addition, the full genomes of the Hawaiian strains of both *P. luteoviolacea*^43^ and *C. lytica* were available^44^, as well as, the genomes for the types strains of both Gram-positive bacteria, allowing a search for the tailocin gene assembly. This study further addresses the question, if tailocins are not transcribed in other inductive bacterial species, what are the mechanisms by which they induce larvae of the tubeworm *Hydroides* elegans to attach to surfaces and metamorphose?

## Results

### Monospecific Bacterial Biofilms

**Figure 1:**
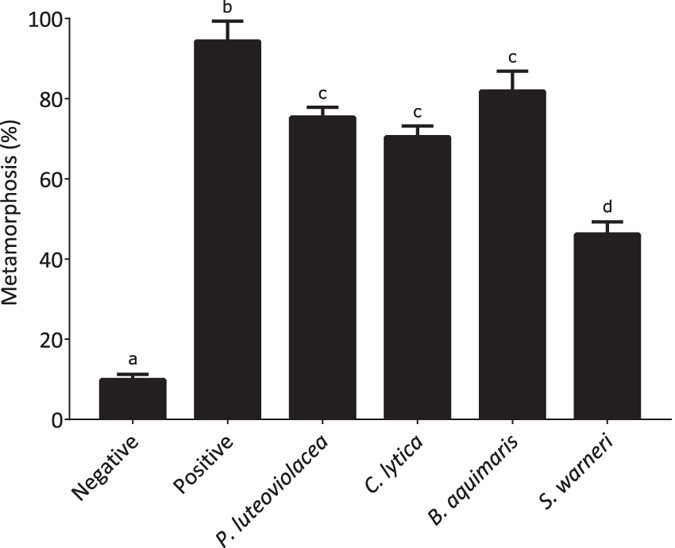
Metamorphosis of *H. elegans* in the presence of monospecific biofilms after 24 hours. A wild type biofilm was used as the positive control and sterile seawater as the negative control.

The settlement responses of competent larvae of *H. elegans* to single-species biofilms of *P. luteoviolacea, C. lytica, B. aquimaris* and *S. warneri* were compared. Metamorphosis was observed for *B. aquimaris* and *S. warneri* with the same inoculation densities as *P. luteoviolacea* (Fig. 1). Consistent with previous results^33^, comparable larval responses were seen only when biofilms of *C. lytica* were inoculated at cell densities approximately 100-fold greater than those of *P. luteoviolacea* (Fig. 1).

### Tailocin assessment

**Figure 2:**
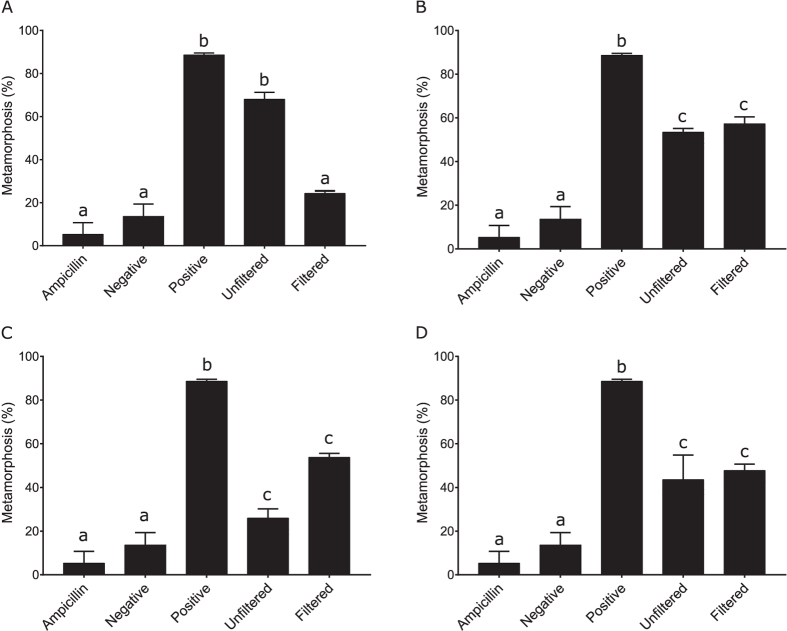
Metamorphic activity of filtered and unfiltered bacteriocin preparations of (**A**) *P. luteoviolacea*, (**B**) *C. lytica*, (**C**) *B. aquimaris* and (**D**) *S. warneri*. Lower case letters represent statistical difference established by Kruskal-Wallis analysis followed by pairwise comparisons with false detection-rate correction.

To determine if the observed biofilm metamorphic activity was due to the presence of tailocins, cultures of all bacterial species were raised for 14 hours and then processed with a method known to produce semi-pure preparations of inductive tailocin clusters in *P. luteoviolacea*^38^. An important aspect of the *P. luteoviolacea* tailocin-induced metamorphosis is that it can largely be removed by filtration (0.22 μm) possibly reflecting retention or disruption of the tailocin aggregates on the filter. Consistent with previous results, the unfiltered portion of the preparation from *P. luteoviolacea* had greater metamorphic activity than the filtered portion (Fig. 2). However, in no replicate bioassay was this residual metamorphic activity completely removed. The metamorphic activity of *C. lytica* and *S. warneri*, however, was unaffected by filtration (Fig. 2). In direct contrast to the filtrate of *P. luteoviolacea*, the filtrate of *B. aquimaris* had inductive activity greater than the unfiltered sample, although this difference was not significant (Fig. 2).

**Figure 3:**
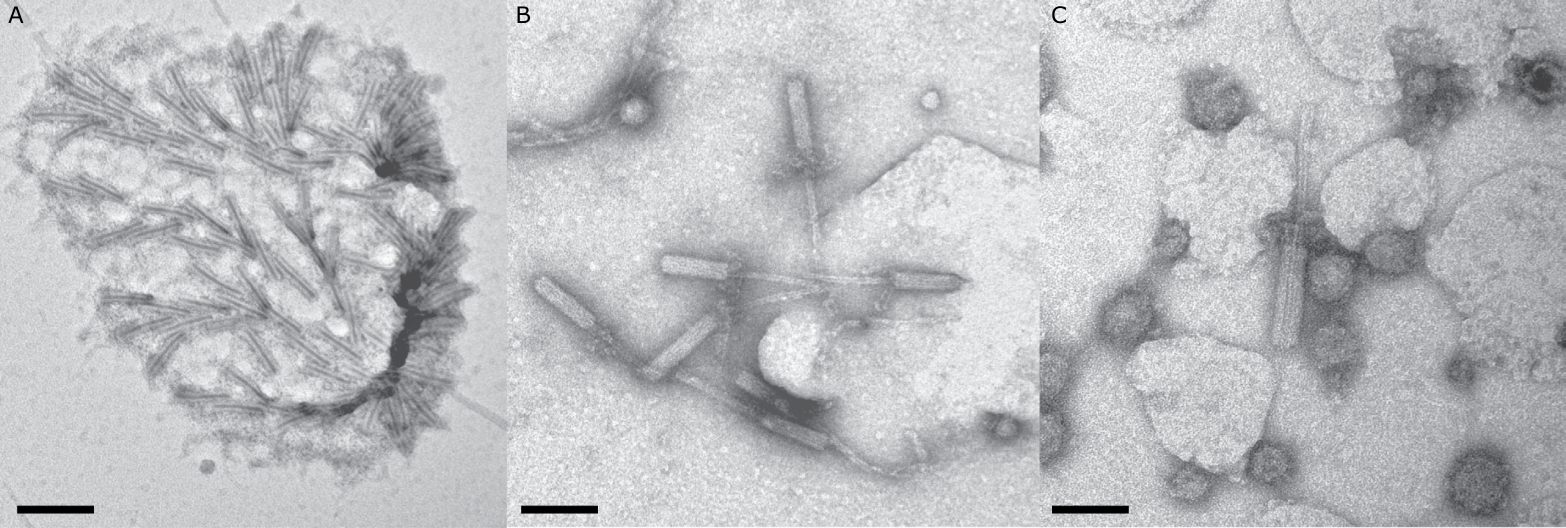
Negatively stained TEM of bacteriocin preparations of *P. luteoviolacea* showing (**A**) Tailocin aggregate, scale bar 175 nm; (**B**) tailocin tubular elements, scale bar 100 nm and (**C**) extracellular vesicles, scale bar 65 nm.

Examination of the filter retentate from *P. luteoviolacea* with TEM revealed the presence of tailocin aggregates (Fig. 3A), as well as numerous individual tubular elements of tailocins (Fig. 3B). These results are consistent with preparation from *P. luteoviolacea* illustrated by Shikuma and colleagues^38^. However, in addition there were also numerous membrane vesicles in these 0.22 μm filter retentates (Fig. 3C).

**Figure 4:**
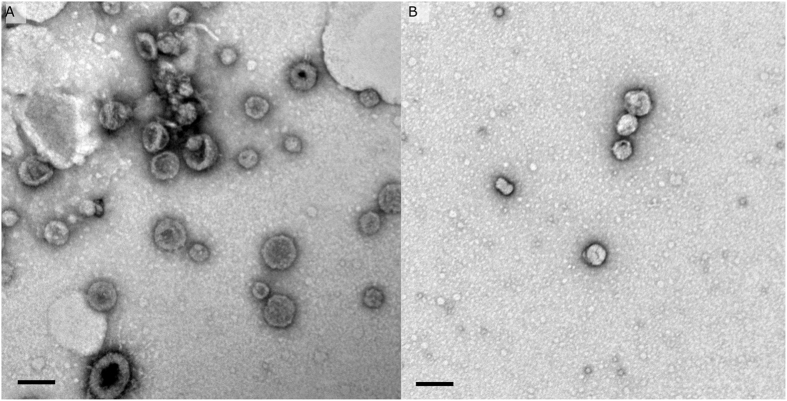
Negatively stained TEM of bacteriocin preparations (**A**) *C. lytica*, scale bar 100 nm and (**B**) *B. aquimaris*, scale bar 60 nm.

Examination of the preparations from *C. lytica* and *B. aquimaris* prepared in the same manner as those from *P. luteoviolacea* revealed none of the tubular tailocin elements, but, instead, showed abundant spherical vesicles ranging in diameter from approximately 40 to 150 nm (Fig. 4). Neither vesicles nor tailocins were apparent on any of the TEM grids prepared with *S. warneri*.

**Figure 5:**
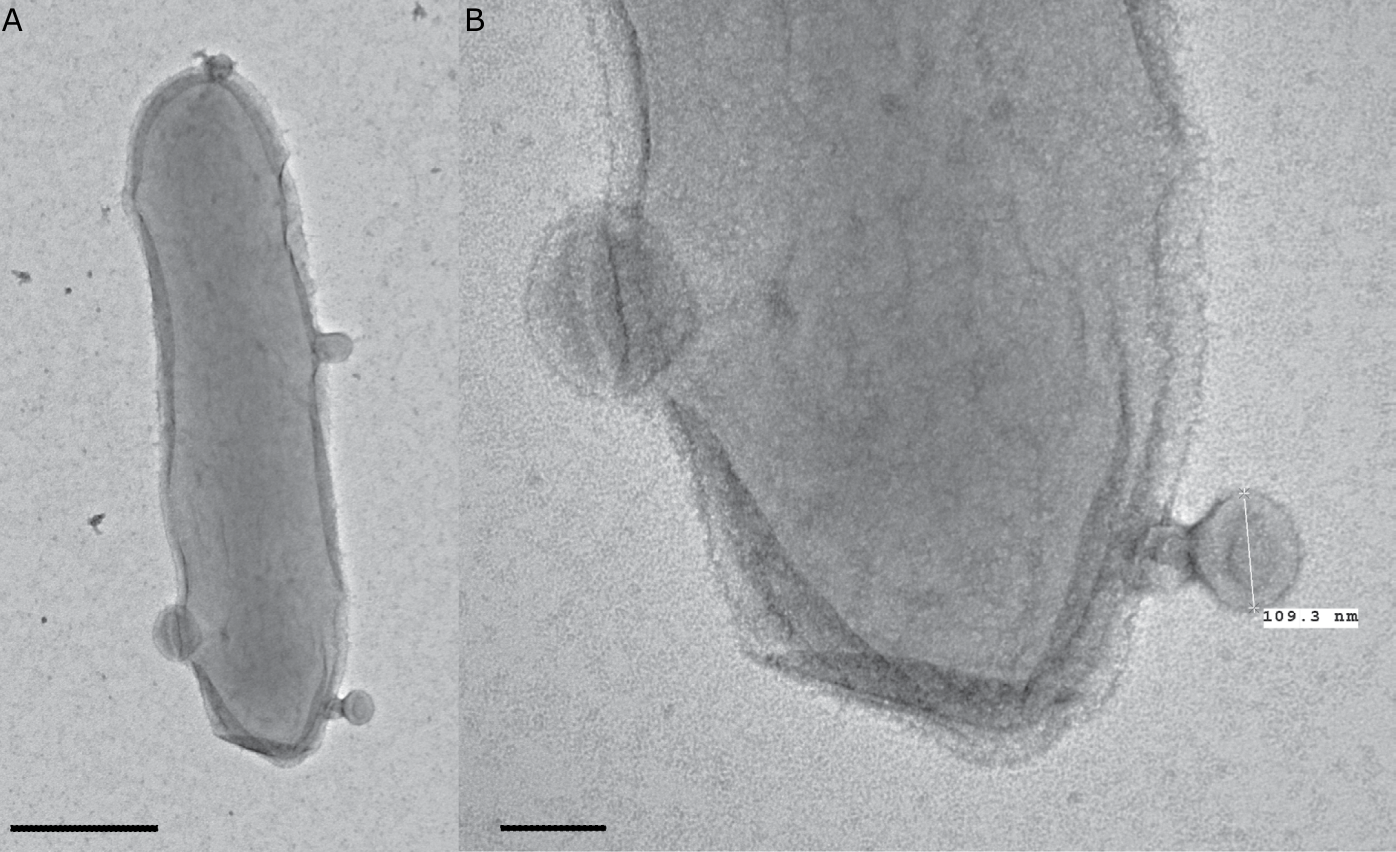
Negatively stained TEM of entire cells of *C. lytica*, showing the formation of outer membrane vesicles (**A**) scale bar 500 nm, (**B**) scale bar 100 nm.

**Figure 6:**
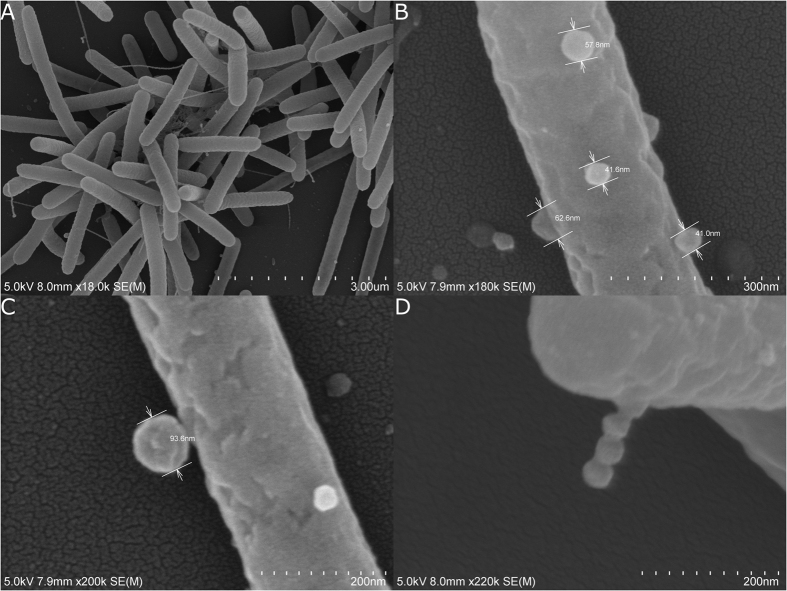
SEM of entire cells of *C. lytica* showing (**A**) entire cells, (**B**,**C**,**D**) different aspects of cells proliferating what appear to be outer membrane vesicles (OMVs).

To further examine the nature of the vesicles observed from cultures of *C. lytica*, entire bacterial cells were fixed and imaged with both TEM (Fig. 5A and B) and SEM (Fig. 6). Both preparations revealed what appear to be vesicles budding from bacterial cells in a manner consistent with the production of outer membrane vesicles or OMVs.

### Extracellular Vesicle (EV) assessment

**Figure 7:**
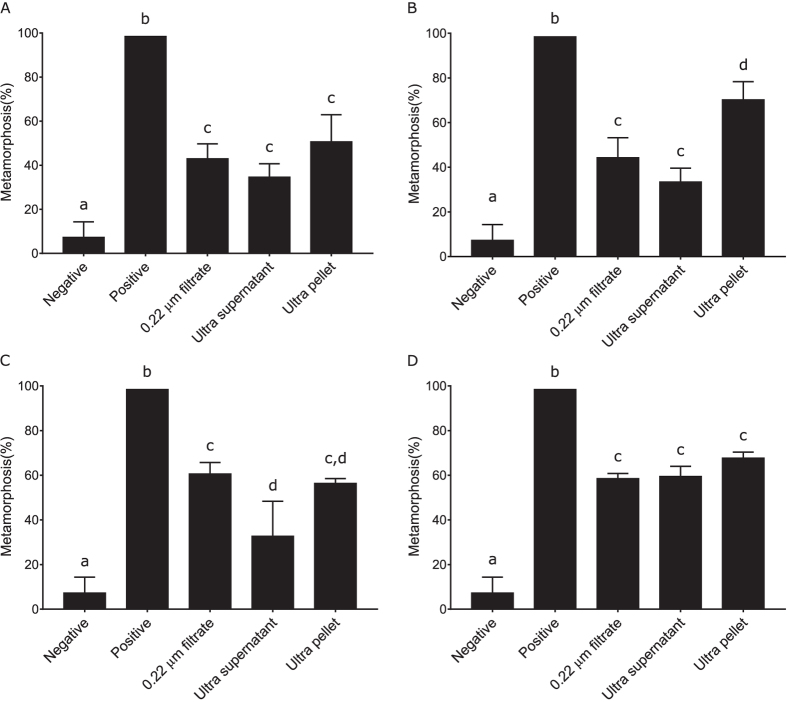
Metamorphic activity of ultracentrifuge MV preparations of (**A**) *P. luteoviolacea*, (**B**) *C. lytica*, (**C**) *B. aquimaris* and (**D**) *S. warneri*. Lower case letters represent statistical difference established by Kruskal-Wallis analysis followed by pairwise comparisons with false detection rate correction.

Ultracentrifuge pellets containing putative EVs were prepared for all four strains of bacteria. These pellets were subjected to bioassay for metamorphosis-inducing capacity and prepared for transmission electron-microscopic examination. Only one strain, *S. warneri*, did not generate a visible pellet by ultracentrifugation of the 0.22 μm-filtered conditioned media; however, a sample was still collected from the appropriate pellet position in the ultracentrifuge tube for this strain. All ultracentrifuge pellets when resuspended in 0.22 μm filtered seawater (FSW) induced settlement in larvae of *H. elegans* (Fig. 7). Interestingly, the small proportion of metamorphic activity that was retained in the filtrate of *P. luteoviolacea* was concentrated into the ultracentrifuge pellet for this strain. This reflects similar patterns for both *C. lytica* and *B. aquimaris* (Fig. 7). In keeping with the lack of visible pellet, metamorphic activity of *S. warneri* was unaffected by ultracentrifugation (Fig. 7).

**Figure 8:**
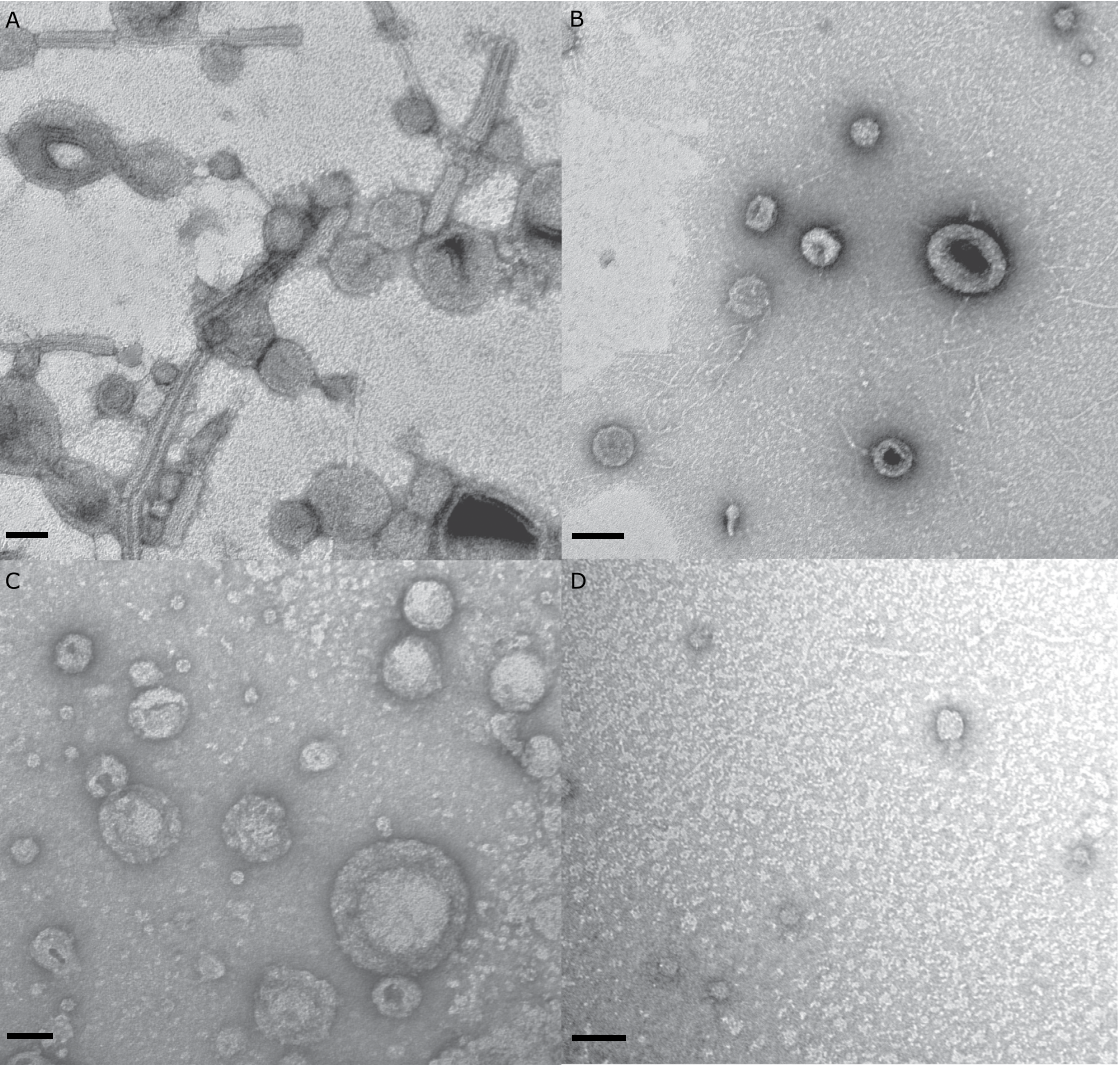
Negatively stained TEM of EV preparations of (**A**) *P. luteoviolacea* scale bar 50 nm, (**B**) *C. lytica* scale bar 100 nm, (**C**) *B. aquimaris* scale bar 50 nm and (**D**) *S. warneri* scale bar 50 nm.

The ultracentrifuge pellet of *P. luteoviolacea* was a deep purple in color and contained both tailocin elements and densely packed vesicles (Fig. 8A). Similar vesicles were observed in the ultracentrifuge pellets of *C. lytica* (Fig. 8B) and *B. aquimaris* (Fig. 8C), but neither pellet contained tailocin elements. The sample taken from the base of the ultracentrifuge tube for *S. warneri* showed numerous small, dense, circular elements approximately 25 nm in diameter (Fig. 8D). TEM examinations, as well as, the absence of colony forming units (CFUs) when the preparations were streaked onto half strength seawater tryptone (1/2FSWT) agar after 48 hours confirmed that these preparations contain no living cells.

### Genome analysis

Basic Local Alignment Search Tool (BLAST) analysis of the genomes of *C. lytica, B. aquimaris* and *S. warneri* for twelve open reading frames (ORFs) that are either required for the induction of metamorphosis of *H. elegans* or implicated in initiation of settlement behavior failed to produce any statistically significant matches to any of the peptides implicated in the induction of metamorphosis by *P. luteoviolacea* (SI Table 1). Only small percentages (40% or less) of the query sequences for the tailocin genes show homology with portions of the genome of *C. lytica* and these peptides are far too small to make up functional tailocins (SI Table 2). BLAST searches for the remaining six ORFs (SI Table 1) recovered no homologues in the genome of *C. lytica* (SI Table 2). Likewise, BLAST analysis against the genome of *B. aquimaris* failed to identify any proteins that showed significant homology to the twelve ORFs from *P. luteoviolacea* (SI Table 3). The inductive ORFs from *P. luteoviolacea* were also absent in the genome of *S. warneri* (SI Table 4), and the results of BLAST analysis showed that over half of these ORFs produced no hits to any genes within the genome of *S. warneri* (SI Table 4).

## Discussion

To understand the process of larval recruitment better, it is critical to learn if all bacterial species that induce settlement in *H. elegans* and other species do so by the same or different mechanisms. Until now, the only reported bacterial elements responsible for this induction were the tightly organized clusters of tailocins termed MACs by Shikuma and colleagues^38^. The research reported here reveals that phage-derived tailocins, singly or in organized clusters, are not universal inducers across inductive bacterial species. The absence of tailocin clusters in the three inductive bacteria *C. lytica, B. aquimaris* and *S. warneri* was conclusively demonstrated by both *in situ* and *in silico* methods. Bioassays revealed metamorphosis-inducing activity in side-by-side preparations from these three species, despite filtration at 0.22 μm, a step that removed most of the inducing capacity of *P. luteoviolacea*^38^. Thus, the inductive elements of the other bacteria were not affected by filtration and were therefore unlikely to consist of aggregate structures larger than 0.22 μm. This evidence was further supported by a lack of tailocin elements, singly or in clusters visible in the TEM images of the other strains. Final confirmation came from the genomic searches and raised the question: of what characteristics of the other biofilm bacterial species are responsible for the induction of metamorphosis in larvae of *H. elegans*.

Instead of phage-derived tailocin structures, the TEM images of *C. lytica* and *B. aquimaris* and *S. warneri* revealed the presence of extracellular vesicles. This is not surprising, because production of extracellular vesicles has emerged as a highly conserved feature among both Gram-positive and Gram-negative bacteria^45^. Extracellular vesicles from both Gram-positive and Gram-negative species have previously been found to fulfill a number of ecological roles by providing a mechanism for cell-to-cell interaction including the transfer of DNA, protein and small signaling molecules^45^,^46^,^47^,^48^,^49^,^50^,^51^,^52^,^53^,^54^. The highly conserved nature of extracellular vesicles combined with their association for communication roles and their presence here in bioactive fractions suggests that such vesicles should be considered for their potential to be a common mechanism of interaction between biofilm bacteria and invertebrate larvae.

Extracellular vesicles have numerous mechanisms of biogenesis^45^,^53^. In *C. lytica* both TEM and SEM of the cellular component revealed vesicles budding from the surfaces of bacterial cells. In Gram-negative bacteria the best studied mechanism of vesicle biogenesis is, similarly, that of budding from the cell surface^55^, providing evidence that the observed vesicles from *C. lytica* are in fact outer membrane vesicles. The observation of budding also allows confirmation that the vesicles observed in the centrifugal preparations are not artifacts of the isolation process, despite a report that vesicles can form spontaneously from cell lysis^56^.

In recent years, extracellular vesicles have also been reported to be produced by Gram-positive bacteria, although the mechanism for their release remains ambiguous^54^. The extracellular vesicles identified in the TEMs from *B. aquimaris* were a heterogeneous mixture from 40 nm to 200 nm in diameter, consistent with previous reports of vesicles isolated from other *Bacillus* species^57^,^58^. It is possible that the diversity of sizes reflects a cargo-sorting mechanism, in which case it may be that the metamorphic induction activity is due to a subset of the vesicles^58^.

In this study, *S. warneri* was the only strain that did not produce a visible pellet by ultracentrifugation; however, small vesicles about 20–25 nm in diameter were apparent in TEM preparations of fluid drawn from the approximate position of the pellet at the bottom of the ultracentrifuge tube. The small size of the vesicles identified here (20–25 nm) represents a technical challenge for isolation, as can be seen by their detection in only one of the two techniques applied in this study. Ultracentrifugation, used in the extracellular vesicle assessment, was powerful enough to concentrate the vesicles for TEM detection. However, the vesicles were not apparent in the tailocin assessment. This is most likely due to differences in the two techniques; the tailocin assessment utilises only low speed centrifugation, and it is possible that there wasn’t sufficient force to concentrate such small vesicles. The possibility that these vesicles were TEM-imaging artefacts was also considered, but deemed unlikely. Vesicles as small as 20 nm have previously been identified from *Staphylococcus aureus* (20 to 100 nm), a prolific producer of extracellular vesicles^59^,^60^. The size of the vesicles in the TEM images of *S. warneri* is also supported by a previous observation that *Staphylococcus* species are capable of producing extracellular vesicles that are smaller than those of other Gram-positive species^61^. Definitive confirmation that the vesicles are the agents inducing metamorphosis of *H. elegans* is hindered, however, as there was no significant difference in metamorphic activity between the pellet position sample and the supernatant.

The identification of extracellular vesicles in tailocin-free fractions that trigger the settlement of *H. elegans* by *C. lytica, B. aquimaris* and *S. warneri* led to a re-examination of their presence and potential role in *P. luteoviolacea*. The original work identifying the tailocins and MACs by Shikuma and colleagues^38^ also showed the presence of vesicles in the TEM images (e.g., Supplemental Figures S3 and S4). Metamorphosis-inducing activity of the tailocin aggregates (MACs) is negatively impacted by filtration, a result that was confirmed here. However, filtration at 0.22 μm is unlikely to impact 20–100 nm-diameter vesicles and is a standard feature in most protocols used to isolate them. In this study, the bioactivity of the filtered *P. luteoviolacea* extracts was never absolutely destroyed (Figs 2A and 6A). Examination of the TEM images of the filtered ultracentrifuge preparations revealed the presence of both individual tailocins and vesicles, hinting at the possibility of a secondary mechanism of interaction with *H. elegans* larvae. MACs were hypothesized to act as metamorphic triggers by puncturing a hole in a cell membrane of the larvae^38^, and it is possible, that although less effective, single tailocins may still fulfil this mechanistic role. The vesicles found in active, cell-free preparations from *P. luteoviolacea* may not be involved in metamorphic induction and might be formed during the explosive cell lysis of MAC release. Alternatively, the reduced level of activity in the filtrate may be due to the presence of the vesicles and the absence of intact tailocin aggregates, and may help to explain why *P. luteoviolacea* is such a strong inducer of settlement in *H. elegans*. Definitive conclusions on this point will likely involve the use of gradient ultracentrifugation to purify the vesicles and separate them from individual tailocins.

The broad size range of vesicles isolated from all four bacterial species studied here raises interesting possibilities regarding their interaction with the larvae of *H. elegans*. Recruitment of sessile marine invertebrates is assured by the capacity of their larvae to settle in response to cues from a variety of bacterial species. Evidence is strong that the very rapid metamorphic events of most invertebrate larvae are neurogenic and initiated from sensory cells on the larval surface^30^,^62^. Neurogenic-electrical spikes have been observed in settlement-induced larvae of a marine gastropod and a sea urchin^63^,^64^, further suggesting involvement of the larval nervous system. Both tailocins and extracellular (or outer membrane) vesicles represent potential mechanisms/vehicles of interaction between bacteria and excitable cells on the surfaces of marine invertebrate larvae. While the tailocin clusters of *P. luteoviolacea* may indeed turn out to be a restricted or isolated mechanism of settlement induction, the widespread nature of extracellular vesicles, as well as, smaller peptide based bacteriocins ideally positions these structures to act as signal systems. Successful recruitment of larvae of *H. elegans* and other sessile marine invertebrates is assured by the capacity of these larvae to be induced to settle by a variety of bacterial species and a variety of induction mechanisms. The important questions that remain are: what are these bacterial mechanisms? Do the various mechanisms stimulate the larval response in similar or dissimilar ways? Is the bacterial induction of settlement in larvae of other invertebrate species likewise tied to extracellular vesicles?

## Methods

### Bioassays

At all stages of separation of biofilms, bacteria and separated bacterial elements, bioassays were conducted with metamorphically competent larvae of *Hydroides* *elegans* as described by Nedved and Hadfield^30^. Briefly, dioecious adult worms collected from docks in Pearl Harbor, HI were induced to spawn by removal from their tubes. Fertilization occurred in the seawater and development proceeded rapidly to a feeding trochophore stage in about two days. Larvae were fed the single-celled alga Isochrysis galbana Tahitian Strain at a concentration of approximately 60,000 cells per ml. The larvae developed to the metamorphically competent nectochaete stage by day five and were employed for up to two days to assay the effects of biofilms including wild, complex natural films, to serve as a positive control, single-species bacterial films, and extracts of single bacterial species. Percent of larvae that metamorphosed was determined at 20–24 hours. Assays were carried out in 24-well plates, with untreated wells containing only FSW serving as negative controls. Significant differences were calculated using Kruskal-Wallis followed by pairwise comparisons with false detection rate (FDR) correction^65^.

### Monospecific Bacterial Biofilms

Monospecific biofilms were generated from cultures of *P. luteoviolacea, C. lytica, B. aquimaris*, and *S. warneri*. Briefly, strains were streaked from −80 °C glycerol stocks onto 1/2FSWt agar plates^66^ and incubated at room temperature for 24 hours. Single colonies were used to inoculate 1/2FSWt broth and incubated for 14 hours at 28 °C with shaking (170 rpm). Cells were pelleted by centrifugation (4,000 *g*, 30 min, 4 °C) and resuspended in 1/10th volume of double-filtered autoclaved seawater. Inoculation cell densities were adjusted to produce 10^8^ cells/ml for all strains except *C. lytica* for which 10^10^ cells/ml were used. A 50 μl aliquot of each culture was streaked onto 1/2FSWt agar plates for CFU counts and allowed 48 hours incubation at room temperature. Biofilm assays were run using 24-well plates (Greiner Bio One, Austria) with a single circular plastic coverslip (12 mm diameter) in the base of each well with four replicates per strain per treatment. Biofilms were formed by adding 500 μl of each treatment per well and allowing 1 hour for cell attachment. After 1 hour the media was aspirated from the wells and the wells washed three times to remove unattached bacteria. Coverslips were then moved to fresh 24 well plates for the bioassays. For confocal microscopy, coverslips were fixed with 4% paraformaldehyde in FSW.

### Isolation of inductive bacterial elements

Cultures of *P. luteoviolacea, C. lytica, B. aquimaris* and *S. warneri* were treated with two separate methods to determine the presence or absence of tailocins or EVs (SI Fig. 1). In order to target potential tailocins, samples were isolated from cultures of *P. luteoviolacea, C. lytica, B. aquimaris* and *S. warneri* following the methods described by Shikuma and colleagues^38^ and referred to as the tailocin assessment. Briefly, −80 °C glycerol stocks of each strain were streaked onto 1/2FSWt agar plates and incubated at 25 °C for 24 hours. Single colonies were then used to inoculate 100 ml 1/2FSWt broth cultures and incubated at 28 °C for 14 hours with shaking (170 rpm). Cells were harvested from the cultures using low speed centrifugation (4,000 *g*, 30 min, 4 °C) and resuspended in 1/10th volume of double autoclaved FSW. Tris buffer was not used at this step as it was found to induce 30% metamorphosis in *H. elegans* (data not shown). This cell suspension was then centrifuged (4,000 *g*, 30 min, 4 °C) and the supernatant carefully aspirated and transferred to a fresh tube. The sample was then centrifuged a final time (4,000 *g*, 30 min, 4 °C) and the supernatant carefully aspirated, transferred to a new tube and kept on ice. To ensure that samples no longer contained viable cells, Ampicillin was added to a final concentration of 100 μg/ml and the samples incubated on ice for 2 hours. After incubation, viability of the samples was confirmed by spread plating 100 μl onto 1/2FSWt agar and incubating for 24 hours at 25 °C. An important feature of the separation method of Shikuma and colleagues^38^ is the demonstration that activity is lost following 0.22 μm filtration. Consequently, an aliquot of each sample was subjected to 0.22 μm filtration (PES, Pall, New York) and the filtrates tested for their ability to induce metamorphosis in larvae of *H. elegans*. In addition, the samples were examined by TEM using negatively stained preparations (see below).

Putative EVs were isolated using a standard ultracentrifugation method^67^ and referred to as the extracellular vesicle assessment. Briefly, −80 °C glycerol stocks of each strain were streaked onto 1/2FSWt agar plates and incubated at 25 °C for 24 hours. Single colonies were then used to inoculate 100 ml 1/2FSWt broth cultures and incubated at 28 °C for 14 hours with shaking (170 rpm). Cells were pelleted from the cultures using low speed centrifugation (4,000 *g*, 30 min 4 °C). The supernatant was collected and treated with 100 μg/ml Ampicillin for 1 hour at 4 °C before undergoing filtration to remove any remaining cells (0.22 μm, PES, Pall, New York). This conditioned media was then subjected to ultracentrifugation to pellet putative EVs (100,000 *g*, 2 hours, 4 °C). All samples were examined by TEM using negatively stained preparations (see below).

### Examination of bacterial preparations with electron microscopy

#### TEM

Bacterial preparations and cell-free preparations were applied to glow-discharged formvar grids, negatively stained with 2% uranyl acetate aqueous solution and air dried. Samples were imaged using a 120 kV Hitachi HT7700 with an AMT XR-41.

#### SEM

Cells from *C. lytica* were fixed with 4% glutaraldehyde with 0.35 M sucrose in 0.1 M sodium cacodylate buffer (pH 7.6). Cells were collected on 0.1 μm polycarbonate filter membranes in Swinex holders, washed with 0.1 M cacodylate buffer, post-fixed with 1% osmium tetroxide in 0.1 M cacodylate buffer then dehydrated through a graded ethanol series. Filters were mounted on aluminum stubs with double-stick conductive carbon tape and coated with gold/palladium in a Hummer 6.2 sputter coater. Specimens were viewed and digital images were acquired with a Hitachi S-4800 Field Emission Scanning Electron Microscope at an accelerating voltage of 5 kV.

### Genomic analysis

BLAST (BLAST + 2.5.0) was used to search within the genome of the *Cellulophaga lytica* HI1 strain (GCA_000750195.1), and the type strains for *B. aquimaris* (GCA_000751975.1) and *S. warneri* (GCA_000332735.1) for homologues of six reading frames (SI Table 1) identified by Shikuma *et al*.^38^,^68^ that were found to encode phage tail elements making up the inductive tailocins of *P. luteoviolacea*^38^ and homologues of six hypothetical proteins involved in the initiation of settlement behavior of *H. elegans*^68^. The searches used standard scoring parameters (Expect threshold: 10, Word size: 6, Matrix: BLOSUM62, Gap Costs: Existence: 11 Extension: 1, Compositional adjustments: Conditional compositional score matrix adjustment), and a low complexity filter was employed.

## Additional Information

**How to cite this article**: Freckelton, M. L. *et al*. Induction of Invertebrate Larval Settlement; Different Bacteria, Different Mechanisms? *Sci. Rep.* **7**, 42557; doi: 10.1038/srep42557 (2017).

**Publisher's note:** Springer Nature remains neutral with regard to jurisdictional claims in published maps and institutional affiliations.

## Supplementary Material

Supplementary Information
