# Supplementary material for: Induction of Invertebrate Larval Settlement; Different Bacteria, Different Mechanisms?
Source: Sci Rep. 2017 Feb 14;7:42557. doi: 10.1038/srep42557 (PMC5307369; doi:10.1038/srep42557)
Supplement: Supplementary Information [file srep42557-s1.pdf]

Supplementary Material for:
Induction of Invertebrate Larval Settlement; Different Bacteria, Different Mechanisms?

Marnie L Freckelton, Brian T Nedved, and Michael G Hadfield*

Kewalo Marine Laboratory, University of Hawaii, Honolulu, 96813, USA
***hadfield@Hawaii.edu**

Experimental Scheme

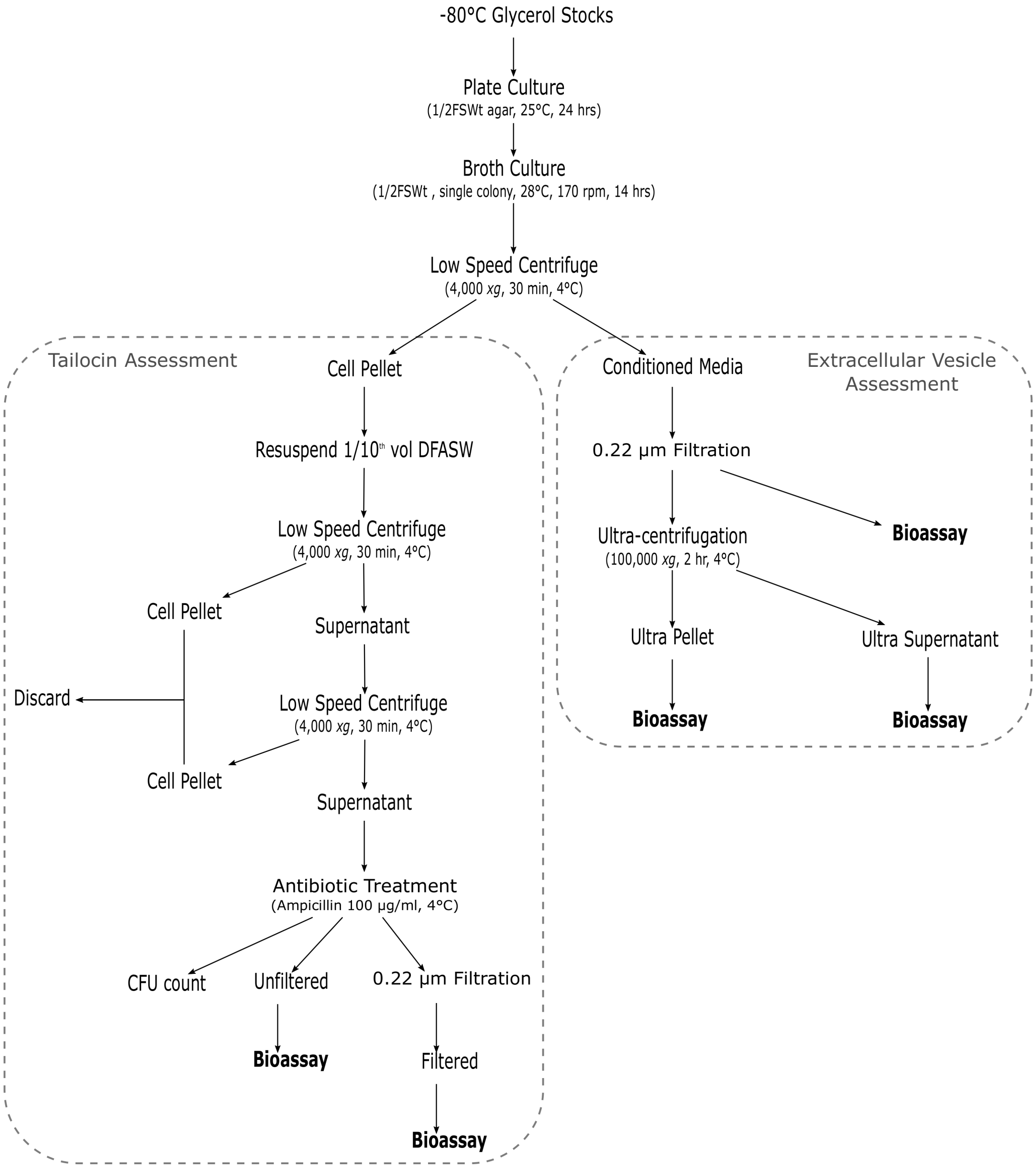

Figure 1. Flow chart of methodology for both tailocin and extracellular vesicle assessments identifying bioassay samples. Methodology was followed in side by side preparations for all four bacterial species (*P. luteoviolacea*, *C. lytica*, *B. aquimaris* and *S. warneri*.

Genomic Assessment Tables

ORF Name	Amino Acid Accession Num.	Protein Name	Length (Amino Acids)
MacS	AHX39703.1	Phage Tail Sheath Protein FI	581
MacT2	AHX39702.1	t4-like Virus Tail Tube Protein	148
MacT1	AHX39701.1	t4-like Virus Tail Tube Protein	153
MacB (ORF1)	AHX39695.1	Baseplate J-like Protein	1070
ORF2	AHX39694.1	Hypothetical Protein	890
ORF3	AHX39693.1	Hypothetical Protein	1671
JF50_12590	AHX39721.1	Hypothetical Protein	139
JF50_12595	AHX39720.1	Hypothetical Protein	135
JF50_12600	AHX39719.1	Hypothetical Protein	131
JF50_12605	AHX39718.1	Hypothetical Protein	747
JF50_12610	AHX39717.1	Hypothetical Protein	496
JF50_12615	AHX39716.1	Hypothetical Protein	943

Table 1. Inductive genes from *Pseudoalteromonas luteoviolacea*. ORF, Open Reading Frame

ORF Name	Query Coverage (%)	Identity (%)	e-Value	Acession Number of Best Amino Acid Hit
MacS	12	30	0.68	WP_051983455.1
MacT2	39	23	4.5	WP_013621992.1
MacT1	37	28	0.64	WP_013619596.1
MacB (ORF1)	9	26	0.49	WP_051983451.1
ORF2	7	28	0.17	WP_051983452.1
ORF3	6	35	0.35	WP_051983429.1
JF50_12590	NA	NA	NA	No Hits
JF50_12595	NA	NA	NA	No Hits
JF50_12600	NA	NA	NA	No Hits
JF50_12605	NA	NA	NA	No Hits
JF50_12610	NA	NA	NA	No Hits
JF50_12615	NA	NA	NA	No Hits

Table 2. Summary of BLAST searches in genome of *C. lytica*. ORF, Open Reading Frame

ORF Name	Query Coverage (%)	Identity (%)	e-Value	Acession Number of Best Amino Acid Hit
MacS	5	35	0.53	WP_044339398.1
MacT2	NA	NA	NA	No Hits
MacT1	13	43	2.3	WP_044339905.1
MacB (ORF1)	4	23	3.7	WP_044337912.1
ORF2	NA	NA	NA	No Hits
ORF3	NA	NA	NA	No Hits
JF50_12590	58	24	4.9	WP_064090927.1
JF50_12595	NA	NA	NA	No Hits
JF50_12600	NA	NA	NA	No Hits
JF50_12605	8	33	2	WP_032088594.1
JF50_12610	6	46	4.5	WP_64091692.1
JF50_12615	5	37	4	WP_64094184.1

Table 3. Summary of BLAST searches in genome of *B. aquimaris*. ORF, Open Reading Frame

ORF Name	Query Coverage (%)	Identity (%)	e-Value	Acession Number of Best Amino Acid Hit
MacS	NA	NA	NA	No Hits
MacT2	NA	NA	NA	No Hits
MacT1	NA	NA	NA	No Hits
MacB (ORF1)	2	3	0.098	WP_002451828.1
ORF2	NA	NA	NA	No Hits
ORF3	3	36	0.48	WP_002451828.1
JF50_12590	15	43	6.2	WP_015364611.1
JF50_12595	NA	NA	NA	No Hits
JF50_12600	NA	NA	NA	No Hits
JF50_12605	2	45	2.1	WP_002466879.1
JF50_12610	NA	NA	NA	No Hits
JF50_12615	6	28	8.7	WP_002466879.1

Table 4. Summary of BLAST searches in genome of *S. warneri*. ORF, Open Reading Frame
